# Combination therapy for treatment of *Pseudomonas aeruginosa* bloodstream infections

**DOI:** 10.1371/journal.pone.0203295

**Published:** 2018-09-20

**Authors:** Sarah Tschudin-Sutter, Nicole Fosse, Reno Frei, Andreas F. Widmer

**Affiliations:** 1 Division of Infectious Diseases and Hospital Epidemiology, University Hospital Basel, University of Basel, Basel, Switzerland; 2 Department of Clinical Research, University Hospital Basel, University of Basel, Basel, Switzerland; Azienda Ospedaliera Universitaria di Perugia, ITALY

## Abstract

**Objectives:**

Management of bloodstream infections (“BSIs”) caused by *Pseudomonas aeruginosa* remains controversial as data supporting the use of definite combination treatment for severe *P*. *aeruginosa* infections remain conflicting. We aimed to determine differences in mortality between patients treated with definite combination therapy and monotherapy in a large 11-year cohort.

**Methods:**

All consecutive patients with *P*. *aeruginosa* BSI hospitalized at the University Hospital Basel, Switzerland, a tertiary academic care center, from January 2003 to December 2013 were included. Pertinent clinical data was assessed. Patients with and without definite combination therapy were compared and hazard ratios for death were calculated.

**Results:**

During the study period, 187 patients with *P*. *aeruginosa* BSI were identified. Definite combination therapy was administered in 42.8% (80/187) of all patients, of which 76% (61/80) received a combination of a betalactam with an aminoglycoside and 24% (19/80) received a combination of a betalactam with a quinolone. The remaining 57.2% (107/187) were treated with betalactam monotherapy. Median treatment duration was 15 days (interquartile range 12–20 days). Mortality was lower in patients receiving definite combination therapy in univariable and multivariable cox regression analyses (HR 0.26, 95% CI 0.11–0.60, p = 0.002 and HR 0.30, 95% CI 0.13–0.71, p = 0.006, respectively), the latter adjusting for age, neutropenia at diagnosis, PITT bacteremia score, and inadequate empirical treatment.

**Conclusions:**

Combination therapy (i.e. betalactam-aminoglycoside or betalactam-quinolone combinations) may improve survival of *P*. *aeruginosa* BSI, independent of potential confounders such as age, neutropenia, PITT bacteremia score, and inadequate empirical treatment.

## Introduction

Mortality of bloodstream infections (BSIs) caused by *P*. *aeruginosa* remains[1–3] and data on survival benefits of different antimicrobial treatment strategies are conflicting[4–15]. Combination therapy for treatment of *P*. *aeruginosa* BSI is administered due to different considerations: first, to cover the known or suspected pathogen while results of identification and susceptibility testing are pending, second, to avoid emergence of resistance during treatment and third, due to possible synergistic activity resulting in better clinical outcome.

It is well-established that the use of combination therapy increases the likelihood of adequate coverage while the results of antimicrobial susceptibility are still pending. However, there is a lack of clinical data supporting the use of two active antibiotic compounds once susceptibility is known.

At our tertiary academic care center, a database of all patients with bloodstream infections has been established. We aimed to determine differences in mortality of patients with *P*. *aeruginosa* BSI treated with definite combination therapy or monotherapy in a large 11-year cohort.

## Patients and methods

### Setting

This study was performed at the University Hospital Basel, a tertiary academic care center admitting more than 30’000 patients annually. The study was approved by the local ethics committee (Ethikkommission Nordwest-und Zentralschweiz EKNZ) as part of the quality assurance program, and therefore informed consent was waived. The STROBE (“Strengthening the Reporting of Observational studies in Epidemiology”) guidelines for reporting of observational studies were adhered to[16].

### Study population

Since 1993, all patients hospitalized with positive blood cultures are prospectively recorded into our in-house BSI database comprising microbiologic, laboratory and clinical data. All adult inpatients with detection of *P*. *aeruginosa* in blood cultures from January 2003 to December 2013 at our institution were included in this study. Baseline characteristics, comorbid conditions, Charlson comorbidity index, systemic inflammatory response syndrome (“SIRS”) criteria, PITT bacteremia score, and antibiotic treatment were assessed.

### Definitions and outcome

*Empirical antimicrobial treatment* was defined as the antimicrobial regimen administered prior to the diagnosis of bacteremia and while identification and susceptibility testing results were still pending. It was considered adequate when the *P*. *aeruginosa* strain identified was susceptible to the antimicrobial compound prescribed.

*Empirical combination therapy* was defined as administration of two antibiotic agents with presumed activity against *P*. *aeruginosa* while results from susceptibility testing were still pending. An empirical combination therapy was deemed to be “adequate” when the activity of the antimicrobials applied was confirmed by subsequent susceptibility testing.

*Definite combination therapy* was defined as administration of two antibiotic agents for at least 48 hours with confirmed activity against the respective *P*. *aeruginosa* isolate as indicated by susceptibility testing.

Neutropenia was defined as a count of neutrophils below 1.0x10^9^/l. Immunosuppressive drugs included high-dose corticosteroids (prednisone equivalent of ≥1 mg/kg body weight in the last 7 days), all types of agents to prevent graft rejection in solid organ or human stem cell/bone marrow transplantation (“HSCT”) such as calcineurin inhibitors or mycophenolate, and to treat autoimmune diseases with monoclonal antibodies such as tumor necrosis factor α inhibitors used in the last 3 months.

Septic shock was defined as sustained hypotension despite adequate fluid replacement requiring vasopressor-support.

The primary outcome was all-cause mortality during hospital stay. The secondary outcome was mortality attributable to *P*. *aeruginosa*-BSI during hospital stay. We understand “attributable mortality” as “total mortality minus the mortality associated with the underlying disease process”. We acknowledge that the term “attributable mortality” is subjected to individual clinical assessment and thus choose to assess both outcomes (all-cause and attributable mortality[17].

### Microbiology

Blood cultures were processed using BacT/ALERT (bioMérieux, Hazelwood, MO) blood culture system throughout the study period. At our institution, at least one pair of aerobic/anaerobic blood cultures (BacT/ALERT FA/FN; bioMérieux) is drawn and cultured, when BSI is suspected. Susceptibility testing is performed following standard procedures using the breakpoints defined by the Clinical and Laboratory Standards Institute (“CLSI”) until 2011 and defined by the European Committee on Antimicrobial Susceptibility testing (“EUCAST”) thereafter. *P*. *aeruginosa* strains were categorized as multidrug-resistant (“MDR”) if non-susceptibility was found in at least one agent in at least three of the following antimicrobial categories: aminoglycosides, antipseudomonal carbapenems, antipseudomonal cephalosporins, antipseudomonal fluoroquinolons, antipseudomonal penicillins plus betalactamase inhibitors, monobactams, phosphonic acids, or polymyxins. Extensively drug-resistance (“XDR”) was defined as non-susceptibility to at least one agent in all but one or two of the above antimicrobial categories as defined elsewhere[18].

Polymicrobial bacteremia was defined as any detection of additional bacteria from the same set of bloodcultures from which *P*. *aeruginosa* was recoverd. Coagulase-negative staphylococci were considered relevant when recovered from at least two bloodcultures.

### Sample size considerations

Based on a prior study reporting mortality of 27% in patients with combination therapy and of 47% in patients with monotherapy for *P*. *aeruginosa* bacteremia[19], a sample size of 176 patients is needed to detect a significant difference in outcome (at the 5% level), assuming superiority and power of 80%.

### Statistical analyses

Patients were categorized as receiving or not receiving adequate empirical treatment and as being treated with or without definite combination therapy. These comparisons were undertaken to (1) identify risk factors for not receiving adequate empirical treatment and (2) to identify possible differences between patients with and without combination therapy, which may act as potential confounders. Chi-square and Fisher’s exact test (where appropriate) were used for comparisons of proportions. For continuous variables, the Shapiro-Wilk test was used to distinguish between normal and abnormal distributions. Normally distributed variables were analyzed using the Student’s t test and non-normally distributed variables using the Mann–Whitney U test. Cox regression analyses were performed to identify variables associated with all-cause mortality and mortality attributable to *P*. *aeruginosa* BSI. In order to identify possible confounding, all variables found to be significant in univariable analyses were included in the multivariable cox regression models. Schoenfeld residuals were calculated to test the proportional hazards assumption. Two-sided, p-values less than 0.05 were considered significant. Analyses were performed using STATA Statistical Software Version 12.0 (Stata Corp., College Station, TX).

## Results

During the study period, 196 patients with *P*. *aeruginosa* BSI were identified. Complete data were missing for 9 patients, so that the final cohort comprised 187 patients. Patients baseline characteristics, clinical features, treatment characteristics and outcome are summarized in Table 1.

**Table 1:** Baseline characteristics of patients with *P*. *aeruginosa* bacteremia.

	n	median	%	IQR
**Demographics**				
Age		66		51–76
Male gender	128		68.5%	
**Underlying conditions**				
Charlson comorbidity index		3		2–4
Intravenous drug use	17		9.1%	
Immunosuppression	89		47.6%	
Solid organ transplant	3		1.6%	
Allogenic stem cell transplant	13		7.0%	
Autologous stem cell transplant	5		2.7%	
Neutropenia at diagnosis	31		16.6%	
**Clinical presentation**				
PITT bacteremia score		1		0–2
Septic shock	20		10.7%	
ICU admission*	71		38.0%	
Mechanical ventilation	13		7.0%	
**Microbiological features**				
Number of positive blood cultures	2			0–2
Polymicrobial sepsis	70		37.4%	
**Origin of bacteremia**				
Urinary tract	42		22.5%	
Unknown	39		20.9%	
Vascular catheter	34		18.2%	
Respiratory tract	31		16.6%	
Abdominal	16		8.6%	
Skin/soft tissue	12		6.4%	
Surgical site infection	6		3.2%	
Bone/joint	4		2.1%	
Pancreaticobiliary	3		1.6%	
**Treatment**				
Adequate empirical treatment	151		80.7%	
Piperacillin/tazobactam	77			
Piperacillin/tazobactam + aminoglycoside	13			
Piperacillin/tazobactam + ciprofloxacin	3			
Piperacillin/tazobactam + aminoglycoside/ciprofloxacin	2			
Cefepime	6			
Cefepime + aminoglycoside	12			
Cefepime + ciprofloxacin	1			
Imipenem	2			
Imipenem + aminoglycoside	2			
Meropenem	12			
Meropenem + aminoglycoside	5			
Meropenem + ciprofloxacin	2			
Ciprofloxacin	12			
Levifloxacin	2			
Inadequate empirical treatment	36		19.25%	
Amoxicillin/clavulanic acid**	21		11.23%	
Ceftriaxone	15		8.02%	
Adequate definite combination treatment	80		42.8%	
Betalactam and fluoroquinolone combination	19			
Betalactam and aminoglycoside combination	61			
Adequate definite single-drug treatment	107		57.2%	
Total duration of antibiotic treatment (days)	15			12–20
**Outcome**				
Death during hospital stay	33		17.7%	
Death attributable to bacteremia	28		15.0%	

ICU: intensive care unit

*Among 71 patients admitted to the ICU, 15 patients were admitted to the ICU within the first few hours after bacteremia, as they presented with septic shock.

** intravenous formulation

As summarized in Table 1, empirical antibiotic treatment was adequate for 151 patients (80.75%). These patients received various empirical treatments, with a relative majority of patients receiving piperacillin/tazobactam (71 patients, 38.0%). Among patients with adequate empirical treatment, all-cause mortality and attributable mortality did not differ between patients receiving active monotherapy (16/111, 14.4% and 12/111, 10.8%) and patients receiving active combination therapy (6/40, 15.0% and 6/40, 15.0%), p = 1.000 and 0.570, respectively. Empirical treatment was not adequate for 36 patients (19.3%) receiving amoxicillin/clavulanic acid (intravenous formulation) (21 patients, 11.2%) or ceftriaxone (15 patients, 8.0%). Among the 40 patients receiving empirical combination therapy, 37 patients remained on definite combination therapy (93%) and three (7%) were switched to monotherapy. Among the remaining 147 patients started on empirical monotherapy, 43 were switched to definite combination therapy, while 104 remained on monotherapy.

Overall we identified 12 MDR strains (6.4%) and one XDR strain (0.5%) in our cohort (Table 2). Their occurrence was evenly distributed throughout the study period. The XDR strain was recovered in 2006, two MDR strains were recovered in 2003, two in 2005, one in 2007, two in 2008, three in 2009 and two in 2012. Among patients with MDR or XDR strains, empirical combination therapy was administred in 5 cases (5/13). The remaining 8 patients received empirical monotherapy, which was inadequate in 5 cases. By allowing a comparison between patients receiving adequate empirical treatment and those receiving inadequate empirical treatment, Table 2 also shows a tendancy concerning the adequacy of empirical treatment for patients having neutropenia at the onset of BSI: these patients are indeed more likely to fall under the “adequate empirical treatment” category. Inadequate empirical treatment was related to both all-cause mortality and mortality attributable to *P*. *aeruginosa* BSI (Table 2).

**Table 2:** Comparisons between patients receiving and not receiving adequate empirical treatment for bacteremia with *P*. *aeruginosa*.

	Patients receiving adequate empirical treatment (n = 151)				Patients receiving inadequate empirical treatment (n = 36)				
	N	median	%	IQR	N	median	%	IQR	p-value
**Demographics**									
Age (years)		66		50–75		67		58–79	0.160
Male gender	99		65.6%		29		80.6%		0.082
**Underlying conditions**									
Charlson comorbidity index		3		2–4		3		2–5	0.159
Intravenous drug use	15		9.9%		2		5.6%		0.412
Immunosuppression	68		45.0%		21		58.3%		0.151
Solid organ transplant	2		1.3%		1		2.8%		0.476
Allogenic stem cell transplant	11		7.3%		2		5.6%		1.000
Autologous stem cell transplant	4		2.6%		1		2.8%		1.000
Neutropenia at diagnosis	31		20.5%		0		0.0%		**0.001**
**Clinical presentation**									
PITT bacteremia score		1		0–2		1		0–2	0.997
Septic shock	16		10.6%		4		11.1%		1.000
ICU admission	53		35.1%		18		50.0%		0.098
Mechanical ventilation	9		6.0%		4		11.11%		0.280
**Microbiological features**									
Number of positive bloodcultures	2			1–4		2		2–3	0.721
Polymicrobial sepsis	55		36.4%		15		41.7%		0.559
MDR *P*. *aeruginosa*	7		4.6%		5		13.9%		0.057
XDR *P*. *aeruginosa*	1		0.66%		0		0.0%		
**Origin of bacteremia**									0.215
Urinary tract	33		21.9%		9		25.0%		
Unknown	27		17.9%		12		33.3%		
Vascular catheter	31		20.5%		3		8.3%		
Respiratory tract	22		14.6%		9		25.0%		
Abdominal	14		9.3%		2		5.6%		
Skin/soft tissue	11		7.3%		1		2.8%		
Surgical site infection	6		4.0%		0		0.0%		
Bone/joint	4		2.7%		0		0.0%		
Pancreaticobiliary	3		2.0%		0		0.0%		
**Outcome**									
Death during hospital stay	22		14.6%		11		30.6%		**0.024**
Death attributable to bacteremia	18		11.9%		10		27.8%		**0.017**

Significant p-values are printed in bold; ICU: intensive care unit, MDR: multidrug-resistant, XDR: Extensively drug-resistance

Definite combination therapy was administered in 42.8% (80/187) of all patients, of which 80% received a combination of a betalactam with an aminoglycoside and 20% (16/80) received a combination of a betalactam and a quinolone. The remaining 57.2% (107/187) were treated with betalactam monotherapy. Median treatment duration was 15 days (IQR 12–20 days). Patients with and without definite combination therapy differed regarding age, while comorbid conditions, immunosuppression, receipt of solid organ or stem cell transplant, need for ICU admission, PITT bacteremia score, and receipt of effective empiric treatment were equally distributed (Table 3).

**Table 3:** Comparisons between patients receiving and not receiving definite combination treatment for bacteremia with *P*. *aeruginosa*.

	Patients with definite combination therapy (n = 80)				Patients with definite monotherapy (n = 107)				
	n	median	%	IQR	n	median	%	IQR	p-value
**Demographics**									
Age (years)		62		48–72		68		57–78	**0.003**
Male gender	49		61.3%		79		73.8%		0.081
**Underlying conditions**									
Charlson comorbidity index		3		2–5		3		1–4	0.233
Intravenous drug use	10		12.5%		7		6.5%		0.201
Immunosuppression	43		53.8%		46		43.0%		0.183
Solid organ transplant	1		1.3%		2		1.9%		1.000
Allogenic stem cell transplant	7		8.8%		6		5.6%		0.563
Autologous stem cell transplant	4		5.0%		1		0.9%		0.166
Neutropenia at diagnosis	17		21.3%		14		13.1%		0.165
**Clinical presentation**									
PITT bacteremia score		1		0–2		1		0–2	0.416
Septic shock	6		7.5%		14		13.1%		0.243
ICU admission	36		45.0%		35		32.7%		0.096
Mechanical ventilation	21		26.3%		23		21.5%		0.488
**Microbiological features**									
Number of positive blood cultures	2			1–3		2		1–4	0.923
Polymicrobial sepsis	24		30.0%		46		43.0%		0.093
MDR *P*. *aeruginosa*	6		7.5%		6		5.6%		
XDR *P*. *aeruginosa*	1		1.3%		0		0.0%		
**Origin of bacteremia**									0.065
Urinary tract	13		16.3%		29		27.1%		
Unknown	19		23.8%		20		18.7%		
Vascular catheter	21		26.2%		13		12.2%		
Respiratory tract	13		16.3%		18		16.8%		
Abdominal	5		6.2%		11		10.3%		
Skin/soft tissue	2		2.5%		10		9.4%		
Surgical site infection	3		3.8%		3		2.8%		
Bone/joint	3		3.8%		1		0.9%		
Pancreaticobiliary	1		1.2%		2		1.9%		
**Treatment**									
Adequate empirical treatment	67		83.8%		84		78.5%		0.454

Significant p-values are printed in bold. ICU: intensive care unit, MDR: multidrug-resistant, XDR: Extensively drug-resistance

**Fig 1:**
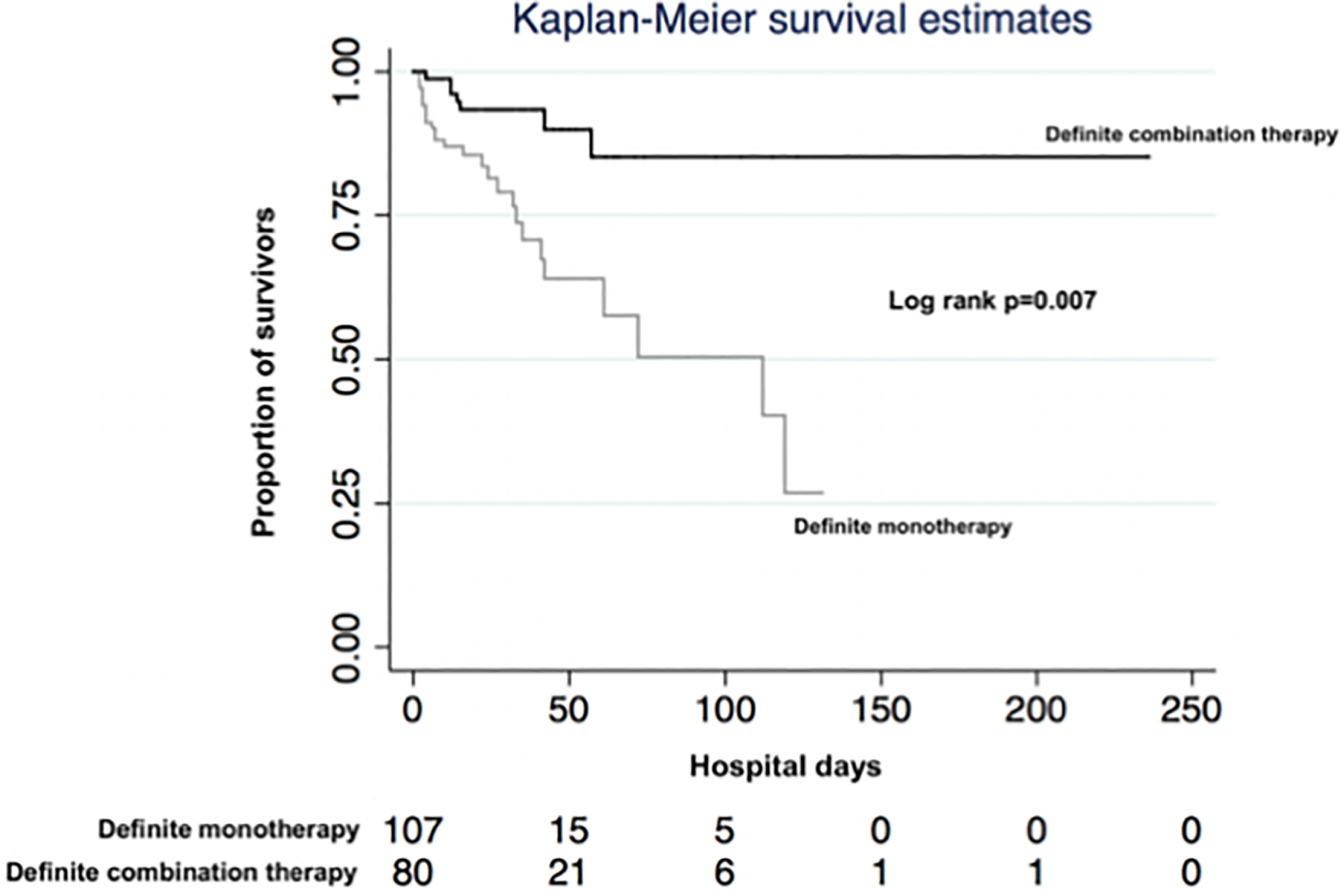
Kaplan-Meier survival curves for patients with and without definite combination therapy. The numbers below the figure represent patients at risk at each time point.

Univariable survival analyses revealed age, neutropenia at diagnosis, PITT bacteremia score, inadequate empirical treatment and definite combination treatment being associated with all-cause mortality (Table 4). The Kaplan-Meier survival curves for patients with and without definite combination therapy are shown in Fig 1, the log rank test revealing a significant difference regarding survival between both treatment strategies (p = 0.007). In multivariable survival analyses, neutropenia at diagnosis, inadequate empirical treatment and non-receipt of definite combination treatment remained independently associated with mortality (Table 4). The test for the proportional hazards assumptions revealed an insignificant p-value (chi square statistic 3.14, p = 0.679) indicating adequate model fit.

**Table 4:** Uni- and multivariable survival analyses (all-cause mortality) for patients with *P*. *aeruginosa* bacteremia.

	Univariable hazards ratio for all-cause mortality			Multivariable hazards ratio for all-cause mortality*		
	HR	95%CI	p-value	HR	95%CI	p-value
**Demographics**						
Age (years)	1.03	1.00–1.05	**0.029**	1.02	0.99–1.05	0.120
Male gender	1.03	0.51–2.12	0.925			
**Underlying conditions**						
Charlson comorbidity index	1.08	0.93–1.26	0.321			
Intravenous drug use	0.30	0.04–2.24	0.243			
Immunosuppression	1.62	0.80–3.31	0.183			
Allogenic stem cell transplant	0.37	0.08–1.61	0.185			
Neutropenia at diagnosis	2.38	1.15–4.96	**0.020**	5.00	2.12–11.65	**<0.001**
**Clinical presentation**						
PITT bacteremia score	1.24	1.06–1.45	**0.008**	1.14	0.97–1.34	0.107
**Microbiological features**						
Polymicrobial sepsis	1.01	0.84–1.21	0.916			
MDR/XDR *P*. *aeruginosa*	1.49	0.56–3.94	0.423			
**Origin of bacteremia**						
Known vs. unknown	1.02	0.44–2.35	0.970			
**Treatment**						
Inadequate empirical treatment	2.51	1.21–5.21	**0.014**	3.32	1.40–7.89	**0.006**
Definite combination treatment	0.26	0.11–0.60	**0.002**	0.30	0.13–0.71	**0.006**

HR: hazard ratio, CI: confidence interval, MDR: multidrug-resistant, XDR: Extensively drug-resistance. Significant p-values are printed in bold

*The multivariable model includes age, neutropenia at diagnosis, PITT bacteremia score, inadequate empirical treatment and definite combination treatment

Multivariable survival analyses regarding death attributable to *P*. *aeruginosa* bloodstream infection as endpoint, revealed similar results: neutropenia at diagnosis (hazard ratio [HR] 3.92, 95% confidence interval [CI] 1.52–10.12, p = 0.005), inadequate empirical treatment (HR 3.27, 95% CI 1.32–8.11, p = 0.011) and non-receipt of definite combination treatment (HR 0.25, 95% CI 0.09–0.68, p = 0.007) were significantly associated with death, the model including age, neutropenia at diagnosis, PITT bacteremia score, inadequate empirical treatment and definite combination treatment. The test for the proportional hazards assumptions revealed an insignificant p-value (chi square statistic 4.65, p = 0.461) indicating adequate model fit.

Six patients died within the first 72 hours after hospital admission, death being considered as attributable mortality in all six cases. As the risk of death is highest within this time period, we performed a sensitivity analyses excluding these six patients. Multivariable survival analyses regarding death attributable to *P*. *aeruginosa* bloodstream infection as endpoint, revealed similar results: neutropenia at diagnosis (HR 3.60, 95% CI 1.22–10.65, p = 0.021), inadequate empirical treatment (HR 4.39, 95% CI 1.55–12.44, p = 0.005) and non-receipt of definite combination treatment (HR 0.30, 95% CI 0.11–0.85, p = 0.024) were significantly associated with death, the model including age, neutropenia at diagnosis, PITT bacteremia score, inadequate empirical treatment and definite combination treatment. The test for the proportional hazards assumptions revealed an insignificant p-value (chi square statistic 4.13, p = 0.531) indicating adequate model fit.

As a significant proportion of patients in our cohort was diagnosed with polymicrobial BSI, we performed a sensitivity analysis by repeating our multivariable survival analysis after exclusion of all patients with polymicrobial bloodstream infection. Neutropenia at diagnosis (HR 4.66, 95% CI 1.53–14.13, p = 0.007), inadequate empirical treatment (HR 4.32, 95% CI 1.34–13.92, p = 0.014) and non-receipt of definite combination treatment (HR 0.20, 95% CI 0.06–0.70, p = 0.011) remained significantly associated with death and the test for the proportional hazards assumptions revealed an insignificant p-value (chi square statistic 4.20, p = 0.521) indicating adequate model fit. Pathogens recovered in addition to *P*. *aeruginosa* are summarized in S1 Table.

## Discussion

In our cohort, administration of definite combination therapy for treatment of *P*. *aeruginosa* BSI was associated with a lower risk for death during hospitalization, independent of important potential confounders, such as age, neutropenia at onset of BSI, PITT bacteremia score, and inadequate empirical treatment. We further could confirm that inadequate empirical treatment is associated with adverse outcome.

Our study adds further credence to the growing body of evidence revealing that inadequate empirical treatment increases mortality in patients with *P*. *aeruginosa* BSI[2,20–22]. Thus, administering two agents in patients with suspected *P*. *aeruginosa* bloodstream infection seems reasonable due to the broader empirical coverage provided by these agents.

The benefits of combination therapy for treatment of *P*. *aeruginosa* BSI once susceptibility results are known is more controversial.

Our findings regarding the survival benefits of definite combination therapy for patients with *P*. *aeruginosa* BSI are supported by an earlier study, demonstrating the superiority of combination therapy as compared to monotherapy in 200 patients with *P*. *aeruginosa* BSI[5]. A benefit for combination therapy in neutropenic patients, but not in non-neutropenic patients was shown in a prospective cohort study including patients not just with *P*. *aeruginosa* but all gram-negative BSIs[9]. Our cohort includes a high proportion of immunocompromised patients possibly explaining the mortality benefit seen in our entire cohort. In a meta-analysis including 17 studies (two randomized trials and 15 observational studies), a survival benefit for patients receiving combination rather than monotherapy could be demonstrated for patients with *P*. *aeruginosa* BSI only[14]. In a recently published Swedish cohort comprising 235 patients definite combination therapy including ciprofloxacin was associated with decreased mortality supporting a beneficial role for combination treatment. Unfortunately, our study is underpowered to draw conclusions regarding ciprofloxacin combination therapy as compared to aminoglycoside combination therapy[23].

In contrast to our findings, a meta-analysis of 64 randomized trials comparing betalactam monotherapy to betalactam-aminoglycoside combination for treatment of sepsis (due to any organism) in immunocompetent patients, revealed no advantages for combination with aminoglycosides regarding survival or development of resistance[13]. This meta-analysis included 426 patients with sepsis caused by *P*. *aeruginosa*. In contrast to our cohort, which has a proportion of 17% of neutropenic patients, neutropenic patients were excluded from this analysis. In addition, the cohort analyzed was more heterogeneous as all types of infections were considered and not just BSIs as in our cohort. In a more recent meta-analysis, no benefit regarding mortality could be identified for patients treated with betalactam-aminoglycoside or betalactam-quinolone combinations as compared to betalactam monotherapy for patients with *P*. *aeruginosa* infections. Patients receiving definite combination therapy, however, tended to have higher clinical cure rates (as measured, *inter alia*, by mortality rates). Of note, again patients with all types of *P*. *aeruginosa* infections–not just BSIs–were included in these analyses, so that a superior effect of combination therapy may have been diluted by including patients with less severe infections[15]. In a large Spanish cohort including 593 patients with *P*. *aeruginosa* BSIs, no benefit regarding survival could be identified for patients treated with combination therapy[1]. In contrast to our cohort, a high proportion of patients initially received inadequate empirical treatment (44% vs. 19%), probably explaining the high mortality rate of 30%, as compared to 18% in our cohort. Possibly, survival benefits of definite combination therapy are difficult to detect in patients receiving inadequate empirical treatment, as this is such a strong predictor for adverse outcome. Furthermore, the proportion of MDR-strains identified in the Spanish cohort was much higher (approximately 30%) as compared to 6.42% in our cohort and only 61 patients finally received aminoglycoside-betalactam or quinolone-betalactam combination therapy as compared to 80 patients in our cohort.

Our study has some important limitations, including its conduction at a single center and its observational study design, as well as its reliance on medical chart review to assess clinical characteristics. We acknowledge that it therefore lacks the quality of a controlled randomized trial to address the question regarding survival benefits of combination therapy for treatment of *P*. *aeruginosa* bloodstream infection. We sought to overcome this shortcoming by carefully assessing our cohort for potential confounding factors, which may be associated with the physician’s choice to administer combination rather than monotherapy and death as our outcome of interest. However, we cannot rule out remaining residual confounding. Due to the retrospective nature of our study, we for example could not adequately collect information on source control in this cohort. We further acknowledge that the sample size of our study is limited, especially when excluding patients with polymicrobial bacteremia. However, the association between definite combination therapy and death remained significant in sensitivity analyses after exclusion of this subgroup excluding inadequate power. Our cohort size of 187 patients meets our set target of 176 patients as determined by power calculation. Our sample size is, however, too small to address certain subgroup analyses or adjustments as for example for different origins of bacteremia.

We further did not assess development of resistance, which may be a possible advantage of combination therapy as compared to monotherapy.

### Conclusions

Our study underscores the importance of adequate empirical treatment in patients with *P*. *aeruginosa* bloodstream infection, thus supporting administration of combination therapy to increase the likelihood of adequate coverage in patients with suspected *P*. *aeruginosa* BSI. Combination therapy (i.e. betalactam-aminoglycoside or betalactam-quinolone combinations) may improve mortality of *P*. *aeruginosa* BSI, independent of potential confounders such as age, neutropenia, PITT bacteremia score, and inadequate empirical treatment.

## Supporting information

S1 Table (DOCX)

S1 Dataset (XLSX)
