# Supplementary material for: Combination therapy for treatment of Pseudomonas aeruginosa bloodstream infections
Source: PLoS One. 2018 Sep 20;13(9):e0203295. doi: 10.1371/journal.pone.0203295 (PMC6147480; doi:10.1371/journal.pone.0203295)
Supplement: S1 Table — (DOCX) [file pone.0203295.s001.docx]

**Supplementary table**

| **Pathogen (in alphabetical order)** | **Number of patients** |
| --- | --- |
| *Bacteroides fragilis* | 7 |
| *Citrobacter* spp. | 3 |
| Coagulase-negative staphylococci | 18 |
| *Eggerthella lenta* | 1 |
| *Enteroccus faecalis* | 6 |
| *Enterococcus feacium* | 3 |
| *Enterobacter cloacae* | 1 |
| *Escherichia coli* | 18 |
| *Klebsiella oxytoca* | 1 |
| *Klebsiella pneumoniae* | 2 |
| *Proteus mirabilis* | 3 |
| *Serratia marcescens* | 3 |
| *Staphylococcus aureus* | 2 |
| *Streptococcus* spp. | 2 |

Pathogens detected in addition to *Pseudomonas aeruginosa* in 70 patients with polymicrobial bacteremia.
